# Impact of physical activity during weekdays and weekends on fat mass among adults: 12-month cohort study

**DOI:** 10.1590/1516-3180.2019.0465.R1.16012020

**Published:** 2020-05-11

**Authors:** Alessandra Madia Mantovani, André Oliveira Werneck, Ricardo Ribeiro Agostinete, Manoel Carlos Spiguel Lima, Jamile Sanches Codogno, Bruna Camilo Turi-Lynch, Rômulo Araújo Fernandes

**Affiliations:** I PhD. Postdoctoral Researcher, Postgraduate Program on Movement Sciences, Universidade Estadual Paulista (UNESP), Presidente Prudente (SP), Brazil; Researcher, Laboratory of Investigation on Exercise (LIVE), Department of Physical Education, Universidade Estadual Paulista (UNESP), Presidente Prudente (SP), Brazil.; II BSc. Master’s Student, Postgraduate Program on Movement Sciences, Universidade Estadual Paulista (UNESP), Presidente Prudente (SP), Brazil; Researcher, Laboratory of Investigation on Exercise (LIVE), Department of Physical Education, Universidade Estadual Paulista (UNESP), Presidente Prudente (SP), Brazil.; III MSc. Doctoral Student, Postgraduate Program on Movement Sciences, Universidade Estadual Paulista (UNESP), Presidente Prudente (SP), Brazil; Researcher, Laboratory of Investigation on Exercise (LIVE), Department of Physical Education, Universidade Estadual Paulista (UNESP), Presidente Prudente (SP), Brazil.; IV PhD. Researcher, Laboratory of Investigation on Exercise (LIVE), Department of Physical Education, Universidade Estadual Paulista (UNESP), Presidente Prudente (SP), Brazil.; V PhD. Assistant Professor, Postgraduate Program on Movement Sciences, Universidade Estadual Paulista (UNESP), Presidente Prudente (SP), Brazil; Lead Researcher, Laboratory of Investigation on Exercise (LIVE), Department of Physical Education, Universidade Estadual Paulista (UNESP), Presidente Prudente (SP), Brazil.; VI PhD. Assistant Professor, Department of Physical Education and Exercise Science. Lander University, Greenwood (SC), United States; Researcher, Laboratory of Investigation on Exercise (LIVE), Department of Physical Education, Universidade Estadual Paulista (UNESP), Presidente Prudente (SP), Brazil.; VII PhD. Associate Professor, Postgraduate Program on Movement Sciences, Universidade Estadual Paulista (UNESP), Presidente Prudente (SP), Brazil; Lead Researcher, Laboratory of Investigation on Exercise (LIVE), Department of Physical Education, Universidade Estadual Paulista (UNESP), Presidente Prudente (SP), Brazil.

**Keywords:** Body composition, Life style, Behavior, Exercise., Physical activity, Weekend warrior, Fat mass, Measurement of physical activity, Adiposity tissue.

## Abstract

**BACKGROUND:**

Physical activity (PA) practices seem to differ between weekdays and weekends and these pattern changes can affect body fat differently. However, previous studies did not assess the mediation effect of weekday and weekend PA on maintenance of body fat using sophisticated statistical models.

**OBJECTIVE:**

To analyze the mediation effect of PA during weekdays and weekends on maintenance of fat mass over a 12-month follow-up.

**DESIGN AND SETTING:**

Longitudinal cohort study (12 months) conducted at a public university in Presidente Prudente, Brazil.

**METHODS:**

A sample of 225 adults (117 females) was used. Body fatness and fat mass were assessed using dual-energy X-ray absorptiometry. PA levels were assessed using a pedometer. The statistical analysis consisted of paired-sample t tests, independent-sample t tests, Pearson correlations and mediation models.

**RESULTS:**

After 12 months, weekend PA had decreased while body composition indicators remained stable (without changes). The correlation between fat mass at baseline and follow-up was high for both sexes (men: 0.966; women: 0.941; P-value = 0.001 for both). Moreover, PA indices were inversely but moderately related to fat mass at baseline and follow-up. Lastly, weekend PA mediated the association between fat mass at baseline and follow-up (P-value < 0.05) by around 2% and 4%.

**CONCLUSION:**

Weekend PA mediated the association between fat mass at baseline and fat mass after one year of follow-up among these adults. Further studies are required to investigate the association between physical activity, body fat and other variables such as dietary patterns and sleep time.

## INTRODUCTION

Independently of age, accumulation of fat mass has adverse effects on human health, mainly because obesity is involved in the genesis of chronic diseases,^1^ and it increases the risk of mortality.^2^ The harmful effects of adiposity on health are well known, but even so, the prevalence of overweight and obesity is still high around the world.^3^ Consequently, medicines, diets and physical activity have been used to change this scenario and its statistics.^4^

Regarding physical activity, studies have shown an independent inverse association between physical activity and body fatness.^5^ Moreover, different intensities and patterns of physical activity seem to present distinct dose-response relationships with body fat, given that activities with higher intensities, such as physical exercise, have a more significant effect on adipose tissue.^6^

However, habitual physical activity among adults constitutes a complex form of human behavior that is strongly affected by social factors. Physical activity guidelines for adults usually adopt recommendations based on five days per week (150 minutes/week, i.e. five days with 30 minutes of activity),^7^ in allusion to the number of working days.

In this kind of approach, the role of physical activity performed during the weekend is underestimated, probably because in our society Saturdays and Sundays are usually a time to restore the energy spent during the regular week. In fact, the weekend could be the perfect moment to engage in moderate-to-vigorous leisure-time physical activity/exercise because it is less influenced by activities that consume time (e.g., job, traffic or children’s schooling). However, it is unclear whether physical activities performed over the weekend have any effect on the health status of adults.^8^

The evidence regarding possible “weekend warrior” effects (a term that was created to characterize individuals who gather much of their physical activity especially through physical exercise on weekends, thus creating irregular patterns of physical activity^8^^,^^9^) is divergent. It has been shown that a session of physical activity during the weekend can be a protective factor against cardiovascular risk,^10^ but on the other hand, the risk of injuries increases.^8^ From the perspective of free-living physical activity, it is also not clear what the effect of weekend physical activity on body composition is. Drenowatz et al.^11^ found that an increase in physical activity levels during weekends over a one-year follow-up was associated with a decrease in fat mass.

However, previous studies did not assess the effect of physical activity during weekdays and weekends on maintenance of body fat using sophisticated statistical models. Multivariate models like linear regression (which were usually adopted in previous studies) identify simultaneous relationships among different variables (direct causal relationships) but do not capture some relevant components of these relationships, such as mediation effects. Mediation analysis enables a more robust understanding of the causal influence of the mediator in the relationship between the exposure and the outcome. Based on previous findings, our hypothesis was that physical activity during weekends could be a protective factor against body fat.^8^^,^^12^

## OBJECTIVE

The aim of this study was to analyze the mediation effect of physical activity during weekdays and weekends on maintenance of fat mass among adults, over one year of follow-up. The initial hypothesis was that physical activity during weekends would be inversely related to fat mass in adults of both sexes.

## METHODS

### Sample

This longitudinal study was developed in the city of Presidente Prudente, which is a medium-sized city of around 200,000 inhabitants that is located in the western region of the state of São Paulo, Brazil. This study combined data from two different cohort studies that were conducted between 2013 and 2015. The research protocols were approved by the local research ethics committee (protocol 173.571/2012 on December 14, 2012; and protocol 349.306/2013 on August 5, 2013), and all subjects signed a consent form. All the evaluations described below were performed at our university’s Laboratory of InVestigation on Exercise (LIVE), and two doctoral students performed the measurements.

The researchers contacted potential participants at the university and at gyms and fitness clubs. The inclusion criteria were that participants should be aged between 30 and 60 years, without any diagnosis of previous cardiovascular complications (e.g. stroke or heart attack), without diabetes complications (amputation or visual problems) and without limitations on physical activity. The sample comprised university staff (professors, administrative staff and gardening/cleaning staff) and members of gyms or fitness centers located in different geographical regions of the city.

 Initially, in the two cohorts together, data-gathering was started among 320 adults, but after accounting for dropouts during the 12 months of follow-up and for missing data (incomplete data on physical activity on any of the seven days), the final sample of this study consisted of 225 participants (n = 107 from the first cohort and n = 118 from the second cohort). Data from the first cohort were collected between 2013 (baseline; n = 122) and 2014 (follow-up; n = 107) and data from the second cohort were collected between 2014 (baseline; n = 198) and 2015 (follow-up; n = 118) using similar inclusion criteria for the two cohorts. Lastly, all procedures (data collection of all variables included in the study) were performed at the first evaluation (baseline) and were repeated 12 months afterwards (follow-up).

### Interview and measurements

The participants attended a face-to-face interview at which they were asked to provide personal data (general information regarding age, sex and ethnicity). On this occasion, anthropometric variables were also measured, using a digital scale for body mass (Filizola, PL-200, to the nearest 0.1 kg) and a fixed stadiometer for height (Sanny, Standard ES2030, to the nearest 0.1 cm). Lastly, from the body mass and height values, the body mass index (BMI; kg/m^2^) was calculated. All these procedures were performed at the first evaluation (baseline) and again 12 months afterwards (follow-up).

### Body composition

Dual-energy X-ray absorptiometry (DXA) (Lunar, DPX-MD model, USA) was used to assess body fatness (percentage values, %) and fat mass (kg). Absolute changes (Δ) and relative changes (Δ%) were calculated for body fatness and fat mass. The DXA scans and definition of lines (regions of interest, ROIs) in the body segments were performed as requested for General Electric Healthcare using a standardized protocol that had been applied in previous studies.^13^^,^^14^ Before the first examination of each day, a trained researcher performed a quality control test. During the scan, the participants remained in the supine position, wearing only light clothing (without shoes). Lastly, the coefficient of variation for this device was determined as 0.66%, through whole-body bone mineral density analysis on 30 individuals who were not involved in this study.

### Physical activity

At the baseline and follow-up, the amount of physical activity (described as steps) was estimated using pedometers (Yamax Digiwalker, SW200 model, Japan). There were no recommendations from researchers regarding physical activity or diet (thus avoiding any kind of interference), but only about the use of pedometers. In accordance with those recommendations, the participants wore the pedometer fixed to one hip for seven consecutive days. The device was taken off only during periods of sleep and during any water-based activities. The participant reported the total number of steps that had been recorded by the device, at the end of every single day of the entire week. Physical activity was divided into activity on weekdays (Monday, Tuesday, Wednesday, Thursday and Friday) and activity on weekends (Saturday and Sunday). The amount of physical activity required for the participant to be classified as “active” was ≥ 7,500 steps on at least five days per week, based on the descriptions of the study by Tudor-Locke et al.^15^ Taking into account both the baseline and the 12-month follow-up, absolute changes (Δ) and the sum of the baseline and follow-up were calculated for physical activity (expressed as numbers of steps).

### Statistical analyses

The descriptive statistics comprised mean values and standard deviations (SD). Comparisons between the two times (baseline and follow-up) were made using a paired-sample t test. Comparisons of changes between men and women at the baseline and follow-up were made using an independent-sample t test. Pearson correlation was used to access correlations between body composition variables and physical activity during weekends and weekdays.

**Figure 1.**
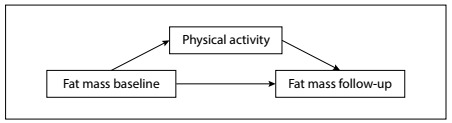
Theoretical model.

Mediation models were performed in accordance with previous recommendations.^12^ Causal mediation was assessed such that it included exposure-mediator interactions, and the total effect was then decomposed into the controlled direct effect and the natural indirect effect, using linear regression models (paramed command).^12^ The analyses were adjusted for sex, chronological age and race. After this, sensitivity analyses were conducted with the aim of estimating potential unmeasured or uncontrolled confounding factors (E-values).^16^ The theoretical model is presented in Figure 1.

All analyses were performed using the STATA software (version 15.1). The significance level (P-value) was set at < 0.05.

## RESULTS

The general characteristics of the sample are described in Table 1 and Table 2. Overall, physical activity during weekends (the number of steps) decreased after one year of follow-up, for both sexes, while body composition indicators remained stable. In addition, at the baseline of the study, 73 participants (32.4%) met the 7,500-step recommendations on at least five days per week, while 152 did not reach the sufficient number of steps (Table 2). Comparison of the changes in physical activity and body composition between the sexes (Table 3) showed that only the sum of physical activity (baseline plus follow-up) was different between men and women (P-value = 0.011).

**Table 1.** Descriptive characteristics of the sample at baseline, according to sex (n = 225)

Variables	Males (n = 108)	Females (n = 117)	P-value
	Mean (SD)	Mean (SD)	
Chorological age (years)	44.34 ± 8.91	47.87 ± 9.11	0.004
Body mass (kg)	84.18 ± 13.76	69.11 ± 13.15	< 0.001
Height (cm)	175.9 ± 7.6	161.3 ± 8.0	< 0.001
Body mass index	27.20 ± 3.92	26.66 ± 5.32	0.382
Waist circumference (cm)*	92.21 ± 10.91	82.23 ± 12.75	< 0.001
Lean soft tissue (kg)	55.99 ± 7.58	37.62 ± 5.27	< 0.001

SD = standard deviation; *n = 215.

**Table 2.** Comparison over time between baseline and follow-up (12 months) regarding body composition variables and percentage of steps during weekends (n = 225)

Variables	Baseline	Follow-up	P-value
	Mean (SD)	Mean (SD)	
Males (n = 108)			
Adiposity			
Body fatness (%)	28.38 ± 8.37	28.11 ± 8.45	0.237
Fat mass (kg)	24.14 ± 9.74	24.08 ± 9.85	0.809
Lean soft tissue (kg)	55.99 ± 7.58	56.20 ± 7.80	0.126
Weekday physical activity			
Number of steps (n)	41,892 ± 20,799	37,110 ± 18,620	0.004
Weekend physical activity			
Number of steps (n)	14,492 ± 8,542	12,152 ± 6,704	0.008
Females (n = 117)			
Adiposity			
Body fatness (%)	40.00 ± 9.36	39.76 ± 9.16	0.503
Fat mass (kg)	27.79 ± 11.00	28.13 ± 11.28	0.337
Lean soft tissue (kg)	37.62 ± 5.27	37.81 ± 5.02	0.338
Weekday physical activity			
Number of steps (n)	38,427 ± 20,783	34,830 ± 20,644	0.006
Weekend physical activity			
Number of steps (n)	11,787 ± 6,712	10,361 ± 6,150	0.005

SD = standard deviation.

**Table 3.** Comparisons of changes (considering 12-month follow-up) regarding body composition and physical activity among men and women (n = 225)

Variables	Male (n = 108)	Female (n = 117)	P-value
	Mean (95% CI)	Mean (95% CI)	
Adiposity			
Absolute change (Δ)			
Body fatness (%)	-0.270 (-0.720 to 0.180)	-0.241 (-0.951 to 0.469)	0.946
Fat mass (kg)	-0.059 (-0.547 to 0.427)	0.340 (-0.359 to 1.040)	0.361
Relative change (Δ%)			
Body fatness (%)	-0.762 (-2.604 to 1.079)	0.280 (-2.334 to 2.895)	0.525
Fat mass (kg)	0.119 (-2.151 to 2.391)	2.328 (-0.988 to 5.644)	0.285
Weekday physical activity (steps)			
Sum (baseline plus follow-up)	79,003 (72,045 to 85,960)	73,258 (66,409 to 80,107)	0.192
Absolute change (Δ)	-4,782 (-7,665 to 1,900)	-3,597 (-6,858 to -337)	0.850
Weekend physical activity (steps)			
Sum (baseline plus follow-up)	26,645 (24,135 to 29,154)	22,148 (20,065 to 24,232)	0.011
Absolute change (Δ)	-2,340 (-3,851 to -828)	-1,425 (-2,528 to -322)	0.673

CI = confidence interval.

Pearson correlations are presented in Table 4. The correlation between fat mass at baseline and follow up was high for both sexes (men: 0.966; women: 0.941; P-value = 0.001 for both). The correlation between physical activity during weekdays and weekends was also significant. Moreover, the physical activity indices were inversely but moderately correlated with fat mass at baseline and follow up, given that the sum of physical activity (on weekends) had the greatest correlation with fat mass, for both sexes: at the baseline (men: -0.381; women: -0.307) and at the follow-up (men: -0.420; women: -0.364).

**Table 4.** Correlation between exposures, mediators and outcomes (n = 225)

	1	2	3	4	5	6	7	8
Males (n = 108)								
1. Fat mass baseline	1							
2. Fat mass follow-up	0.966**	1						
3. Physical activity baseline (weekends)	-0.339**	-0.369**	1					
4. Physical activity follow-up (weekends)	-0.316*	-0.354**	0.481**	1				
5. Physical activity sum (baseline plus follow-up) (weekends)	-0.381**	-0.420**	0.895**	0.822**	1			
6. Physical activity baseline (weekdays)	-0.400**	-0.387**	0.641**	0.491**	0.667**	1		
7. Physical activity follow-up (weekdays)	-0.332*	-0.344**	0.419**	0.681**	0.619**	0.711**	1	
8. Physical activity sum (baseline plus follow-up) (weekdays)	-0.397**	-0.396**	0.579**	0.628**	0.696**	0.933**	0.916**	1
Females (n = 117)	1	2	3	4	5	6	7	8
1. Fat mass baseline	1							
2. Fat mass follow-up	0.941**	1						
3. Physical activity baseline (weekends)	-0.266*	-0.338**	1					
4. Physical activity follow-up (weekends)	-0.277*	-0.304*	0.564**	1				
5. Physical activity sum (baseline plus follow-up) (weekends)	-0.307*	-0.364**	0.895**	0.873**	1			
6. Physical activity baseline (weekdays)	-0.319*	-0.335**	0.578**	0.507**	0.615**	1		
7. Physical activity follow-up (weekdays)	-0.336**	-0.339**	0.460**	0.797**	0.702**	0.631**	1	
8. Physical activity sum (baseline plus follow-up) (weekdays)	-0.362**	-0.373**	0.575**	0.721**	0.729**	0.904**	0.902**	1

The numbers (1 to 8) in the first line mean each variable of exposures, mediators and outcomes inserted in the correlation model (shown in numerical order in column 1). *P < 0.05; **P < 0.001.

Models of the mediation by physical activity indicators on the association between fat mass at baseline and fat mass at follow-up are presented in Table 5. Physical activity during weekends (baseline, follow-up and the sum of baseline and follow-up) mediated the association between fat mass at baseline and follow-up (P-value < 0.05); the percentage of mediation was between 2% and 4%, and the potential influence of unmeasured confounders (E-values) ranged from 1.13 to 1.17. On the other hand, physical activity during weekdays did not mediate the association between fat mass at baseline and follow-up.

**Table 5.** Mediation models for different physical activity levels during weekdays and weekends and the association with fat mass at baseline and fat mass at follow-up (one year)

	Total effect β (95% CI)	Controlled direct effect β (95% CI)	Natural indirect effect β (95% CI)	E-value RR
Weekdays				
Baseline physical activity	0.972 (0.930 to 1.014)	0.963 (0.919 to 1.007)	0.009 (-0.007 to 0.023)	1.097
Follow-up physical activity	0.972 (0.930 to 1.014)	0.962 (0.919 to 1.006)	0.009 (-0.004 to 0.023)	1.102
Baseline + follow-up physical activity	0.972 (0.930 to 1.014)	0.961 (0.916 to 1.005)	0.011 (-0.005 to 0.027)	1.112
Weekends				
Baseline physical activity	0.972 (0.930 to 1.014)	0.951 (0.908 to 0.994)	0.021 (0.005 to 0.036)	1.158
Follow-up physical activity	0.972 (0.930 to 1.014)	0.957 (0.914 to 1.000)	0.015 (0.001 to 0.028)	1.130
Baseline + follow-up physical activity	0.972 (0.930 to 1.014)	0.948 (0.904 to 0.991)	0.024 (0.008 to 0.041)	1.174

Adjusted for chronological age, sex and race.

CI = confidence interval; RR = risk ratio.

## DISCUSSION

In the present study, the aim was to investigate the effect of physical activity during weekends and weekdays on maintenance of fat mass among adults, over a one-year follow-up. The main result found was that physical activity during weekends, but not weekdays, partially mediated the association between fat mass at baseline and fat mass after one year of follow-up, given that a higher amount of physical activity was associated with reduction in fat mass.

Obesity during adulthood presents high stability, even in long-term follow-up studies.^17^ This may be due to the biology of fat cells, which have a characteristic of stability over several hyperplasia events.^18^ In this regard, understanding the real impact of each factor on adiposity during adulthood is important, in order to support formulation of possible intervention programs. With this in mind, several factors can change the trajectory of body fatness during adulthood, especially behavioral factors, such as dietary patterns^19^ and physical activity.^20^ Among the domains of physical activity, leisure-time is most associated with reduction of body fat and improvements in metabolic profile, especially when exercise is included,^6^ given that this generally has sufficient intensity to promote health gains.

We found that even though physical activity during weekends (but not weekdays) had a low effect, it partially mediated the association between fat mass at the baseline and at the follow-up, thus promoting a protective effect (reduction of body fat). This is an important issue, given that only physical activity performed during weekends, with a relatively small follow-up period, was a high stable factor (body fat). Therefore, physical activity during weekends can be a protective factor, especially if other healthy behaviors during weekends are taken into consideration. Energy intake, especially through carbohydrates and fat, is usually high during weekends.^21^ Therefore, a greater level of physical activity could attenuate the association between poorer dietary patterns and obesity. Another factor that can contribute to obesity on weekends relates to sleep patterns: people generally sleep for longer times than on weekdays.^22^ The reduction of energy expenditure during weekends could be attenuated by a greater level of physical activity.

From another point of view, the correlation between physical activity during weekdays and weekends was high, such that the subjects with higher physical activity levels during weekends also had higher physical activity during weekdays. Therefore, the homogeneity of physical activity levels during weekdays could also explain why there was an association with physical activity during weekends but not with physical activity during weekdays. In addition to the well-recognized fact that physical activity may be attenuated during weekends, given the level of occupation during weekdays, weekend physical activity could thus be an alternative, bearing in mind that even if physical activity levels during weekends might not meet the recommendations, a greater level of physical activity during weekends can be a protective factor against obesity and all-cause mortality.^10^^,^^11^

This is a special issue in relation to our sample, which was composed of university staff who were working for eight hours per day during the week. In this regard, our study shows clear practical implications, through confirming that high levels of physical activity during weekends seem to be a good strategy for reducing body fat. However, these data should be interpreted with caution. Even with the evidence showing that greater levels of physical activity during weekends can be considered to be a protective factor against obesity, shown in our study, and against mortality, shown in previous studies,^10^^,^^23^ some types of activity may be dangerous when done only during weekends. This is especially so in relation to high-intensity activities, which increase the chance of injuries.^24^

Our study has limitations that need to be pointed out. Considering the missing data and dropout rate, the sample of our study was reduced in size by 29.7% between the baseline and follow-up measurements. A larger sample size would have given rise to lower risk of bias of the results. The measurement of physical activity using a pedometer was objective, but it only took into account steps and did not assess the intensity of physical activity.^25^ Moreover, our study did not include potential confounders such as sleep and dietary patterns, which could be potential moderators in the models.^21^ In the analyses, although we made adjustments according to sex, the sample was not divided into subgroups according to sex, to be analyzed.

On the other hand, we made use of a good indicator of body fat levels (DXA)^26^ and presented data from a 12-month follow-up regarding the effect of objectively measured physical activity on body fat among adults. We assume that this was a point of strength in this study.

## CONCLUSION

In summary, physical activity during weekends partially mediated the association between fat mass at baseline and fat mass after one year of follow-up among adults. Furthermore, future studies should investigate the joint associations between dietary patterns, sleep time, physical activity during weekends and body fat. Considering the clinical implications, stimulation of habitual physical activity (i.e. increasing the number of steps per day) is a simple, cheap and efficient tool for reducing the fat mass over one year, especially on weekend days.
